# Selective Elimination of Human Induced Pluripotent Stem Cells Using Medium with High Concentration of L-Alanine

**DOI:** 10.1038/s41598-018-30936-2

**Published:** 2018-08-20

**Authors:** Takunori Nagashima, Kazunori Shimizu, Ryo Matsumoto, Hiroyuki Honda

**Affiliations:** 1 Department of Biomolecular Engineering, Graduate School of Engineering, Nagoya University, Nagoya, Japan; 2 Innovative Research Center for Preventive Medical Engineering, Nagoya University, Nagoya, Japan

## Abstract

Human pluripotent stem cells, including human induced pluripotent stem cells (hiPSCs), serve as highly valuable sources for both cell-based therapies and basic research, owing to their abilities to self-renew and differentiate into any cell type of the human body. However, tumorigenic risks of residual undifferentiated stem cells limit the clinical application of hiPSCs, necessitating methods to eliminate undifferentiated hiPSCs from differentiated cells. Here, we found that undifferentiated hiPSCs were more sensitive to the treatment with a medium supplemented with high concentration of L-alanine than human fibroblasts (hFBs), human skeletal muscle cells (hSkMCs), hiPSC-derived cardiomyocytes (iCMs) or hiPSC-derived fibroblast-like cells (iFLCs), which were used as differentiated cells. Undifferentiated hiPSCs co-cultured with differentiated cells were selectively eliminated following treatment. In addition, we found that the medium supplemented with high concentration of D-alanine or β-alanine also induced cell death of hiPSCs and the treatment at 4 °C didn’t induce cell death of hiPSCs. The cell death induced would be associated partly with high osmotic pressure of the medium supplemented with L-alanine. As L-alanine is a component of proteins in human body and popular ingredient of cell culture media, treatment with high concentration of L-alanine may be useful for eliminating tumorigenic residual hiPSCs for stem cell-based therapies.

## Introduction

Human pluripotent stem cells (hPSCs) such as human embryonic stem cells (hESCs)^1^ and human induced pluripotent stem cells (hiPSCs)^2^ serve as highly valuable sources for both cell-based therapies and basic research, owing to their abilities to self-renew and differentiate into any cell type of the human body. However, there are several limitations associated with the use of hESCs in cell-based therapy. The first issue is the immune incompatibility between the donor cells and the recipient. The second issue is ethical constraints, as the embryo dies during the isolation of hESCs^3^. These constraints could be overcome with the use of hiPSCs, which may be directly generated from various somatic cells. Thus, hiPSCs may serve as promising materials for regenerative therapy. Nevertheless, their ability to undergo unlimited self-renewal and pluripotent differentiation makes hiPSCs tumorigenic after transplantation. Therefore, complete differentiation or selective elimination of residual undifferentiated cells is essential for the clinical application of these derivatives^4,5^.

Several strategies have been reported to promote the selective removal of hiPSCs from a population of differentiated cells, such as the introduction of suicide genes into hiPSCs^6^, application of plasma-activated medium^7^, use of hiPSC-specific cytotoxic antibodies^8^ or lectin^9^, alteration of cell culture conditions^10^, and cell sorting using antibody against hiPSC surface antigens^11^ and chemical inhibitors^12,13^. However, none of these methods have reached the level of clinical application for regenerative therapy, owing to the cost, throughput, specificity, and effect of residual agents^14^. Therefore, a novel strategy for the elimination of undifferentiated hiPSCs with distinct elimination mechanisms is requisite.

We aimed to establish a novel strategy to eliminate undifferentiated hiPSCs using components which are generally contained in cell culture media, such as ions, sugars, and amino acids. In the present paper, we proposed a novel method to eliminate undifferentiated hiPSCs by adjusting amino acid concentration in cell culture medium. As amino acids are general organic and monomeric components of proteins in human body and form popular ingredients of cell culture media, the use of amino acids as agents to eliminate undifferentiated hiPSCs may be implemented as a low-cost, simple, easy, and safe methodology. Herein, we used L-alanine and investigated whether hiPSCs may be selectively eliminated following their treatment with a medium supplemented with high concentration of L-alanine.

## Results

### Differential sensitivities of undifferentiated and differentiated cells toward medium supplemented with L-alanine

**Figure 1:**
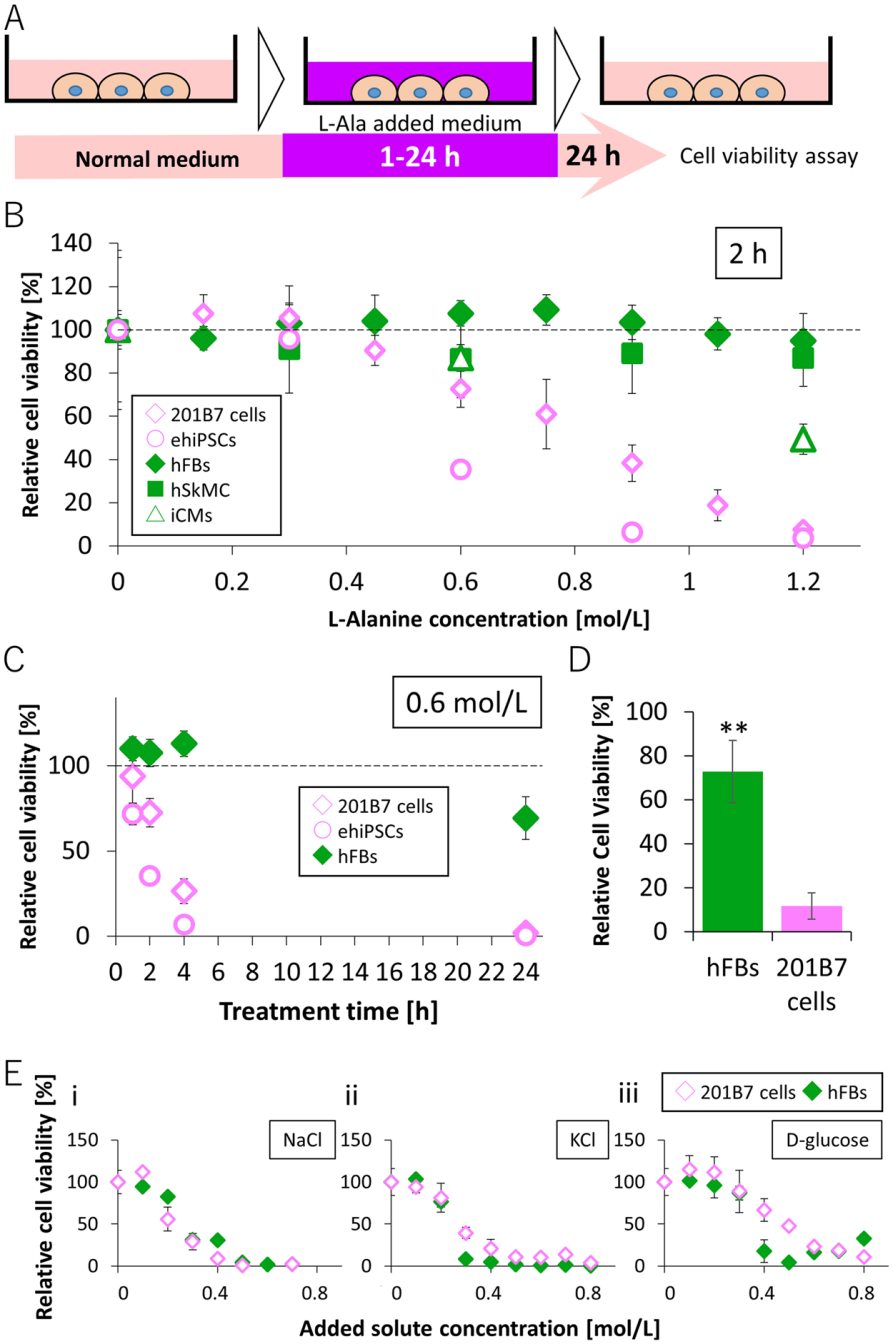
Differential sensitivities of undifferentiated and differentiated cells in medium supplemented with L-alanine. (**A**) Schematic representation of the protocol for the treatment with medium supplemented with L-alanine. Cells were cultured in normal medium and treated with 0 to 1.2 mol/L L-alanine (supplemented in the medium) for 0 to 24 h. The medium was replaced with the normal medium. After 24 h cultivation, cell viability was evaluated. (**B**) Viability of cells treated with medium supplemented with L-alanine for 2 h. Unfilled diamond (pink): 201B7 cells, unfilled circle (pink): ehiPSCs, filled diamond (green): hFBs, filled square (green): hSkMCs, unfilled triangle (green): iCMs. (**C**) Viability of cells treated with 0.6 mol/L L-alanine for 1, 2, 4, and 24 h. Unfilled diamond (pink): 201B7 cells, unfilled circle (pink): ehiPSCs, filled diamond (green): hFBs. (**D**) Viability of cells treated with medium supplemented 1.2 mol/L L-alanine for 2 h in suspension culture condition. The means ± SD of three or four experiments are shown; **p < 0.01. (**E**) Viability of cells treated with medium supplemented with NaCl (i), KCl (ii), and D-glucose (iii) for 2 h. Unfilled diamond (pink): 201B7 cells, filled diamond (green): hFBs.

To investigate the selective removal of hiPSCs from differentiated cells by the high–L-alanine medium, we used two types of hiPSCs, 201B7 hiPSCs (201B7 cells) and an hiPSC line derived by episomal system (ehiPSCs), along with normal human dermal fibroblasts (hFBs), human skeletal muscle cells (hSkMCs) and hiPSC-derived cardiomyocytes (iCMs) as differentiated cells. As shown in Fig. 1A, the cells were incubated in a medium supplemented with L-alanine at various concentrations (0–1.2 mol/L) or treatment times (1–24 h). The medium was replaced with a normal medium and the relative cell viability was measured after 24 h.

The effects of L-alanine concentration were investigated by incubating cells with the medium containing various concentrations of L-alanine for 2 h (Fig. 1B). The viability of both hiPSCs, 201B7 cells and ehiPSCs decreased with an increase in L-alanine concentration, and reached 7.5 ± 1.3% and 3.7 ± 0.7% respectively at 1.2 mol/L concentration of L-alanine. On the other hand, no decrease in the viability of hFBs and hSkMCs were observed (94.9 ± 12.5% for hFBs and 87.0 ± 13.1% for hSkMCs) (Fig. 1B). Although the viability of iCMs slightly decreased along with the increase of the L-alanine concentration, viability of iCMs at 1.2 mol/L concentration of L-alanine, 49.4 ± 6.9%, was significantly higher than that of undifferentiated iPSCs, 201B7 cells and ehiPSCs (p < 0.01). We observed that 201B7 cells treated with 1.2 mol/L L-alanine for 2 h gradually stained positive for propidium iodide (PI) after returning to the normal medium.

In addition, we examined the effects of treatment time by incubating cells with the medium supplemented with L-alanine at 0.6 mol/L concentration for 1, 2, 4, and 24 h (Fig. 1C). The viability of hiPSCs, 201B7 cells and ehiPSCs, drastically decreased even after 2 or 4 h treatment (Fig. 1C). In contrast, the viability of hFBs failed to decrease at 1, 2, and 4 h and showed a small decrease (69.3 ± 1.3%) at 24 h treatment.

We investigated whether the sensitivity difference was observed for suspension culture as well as two-dimensional (2D) adhesive culture condition. The viability of 201B7 cells in suspension culture decreased to 11.8 ± 6.0% following treatment with 1.2 mol/L L-alanine for 2 h, whereas that of hFBs was 72.9 ± 14.2% (Fig. 1D).

We presumed that the drastic change in osmotic pressure was one of the reasons for the selective elimination of undifferentiated cells. Therefore, we examined the effects of NaCl, KCl, and D-glucose, which are representatives of ions and sugars contained in cell culture medium, on the viability of hiPSCs and hFBs. However, high concentration of NaCl, KCl, or D-glucose did not eliminate undifferentiated hiPSCs selectively (Fig. 1E).

These results suggest that hiPSCs are more sensitive to medium supplemented with high concentration of L-alanine than differentiated cells. In subsequent experiments, we treated cells with the medium supplemented with L-alanine at 1.2 mol/L concentration for 2 h.

### Selective elimination of undifferentiated cells by L-alanine added in the medium

**Figure 2:**
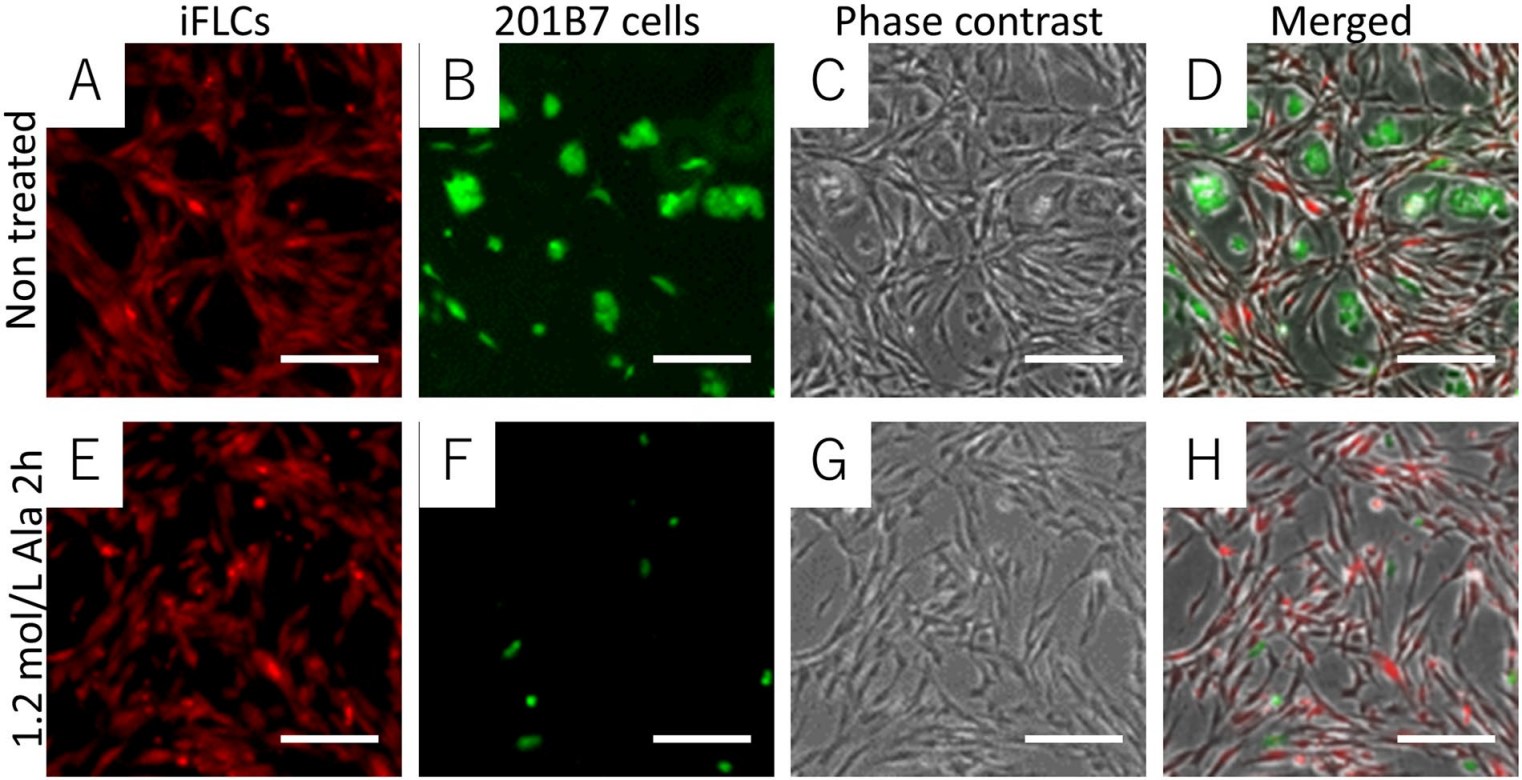
Selective elimination of hiPSCs co-cultured with hiPSC-derived fibroblast-like cells (iFLCs). (**A**–**D**) Images of non-treated cells. (**E**–**H**) Images of the cells treated with medium supplemented with 1.2 mol/L L-alanine for 2 h. (**A** and **E**) Images of iFLCs stained with Cell Tracker Orange. (**B** and **F**) Images of 201B7 cells stained with Cell Tracker Green. (**C** and **G**) Phase-contrast images. (**D** and **H**) Merged images of (**A**–**C** and **E**–**G**). Scale bar, 50 µm.

We investigated whether L-alanine selectively eliminated undifferentiated 201B7 cells when co-cultured with differentiated cells. First, we used hiPSC-derived fibroblast-like cells (iFLCs) differentiated from 201B7 cells (Fig. S1)^15^. Fluorescently-labeled hiPSCs and iFLCs were seeded in wells of a multi-well plate and treated with 1.2 mol/L L-alanine supplemented medium for 2 h (Fig. 2). Following treatment, most hiPSC colonies were eliminated from the well, whereas iFLCs were retained (Fig. 2).

Second, we quantitatively tested whether L-alanine selectively eliminated undifferentiated 201B7 cells co-cultured with hFBs. Fluorescently-labeled hiPSCs (green) and hFBs were seeded in a multi-well plate at different ratios (initial 201B7 cells to hFBs ratios were 1:1, 1:2, and 1:4) and treated with medium supplemented with 1.2 mol/L L-alanine for 2 h. After treatment, the existing ratios of residual 201B7 cells were measured using flow cytometer.

**Figure 3:**
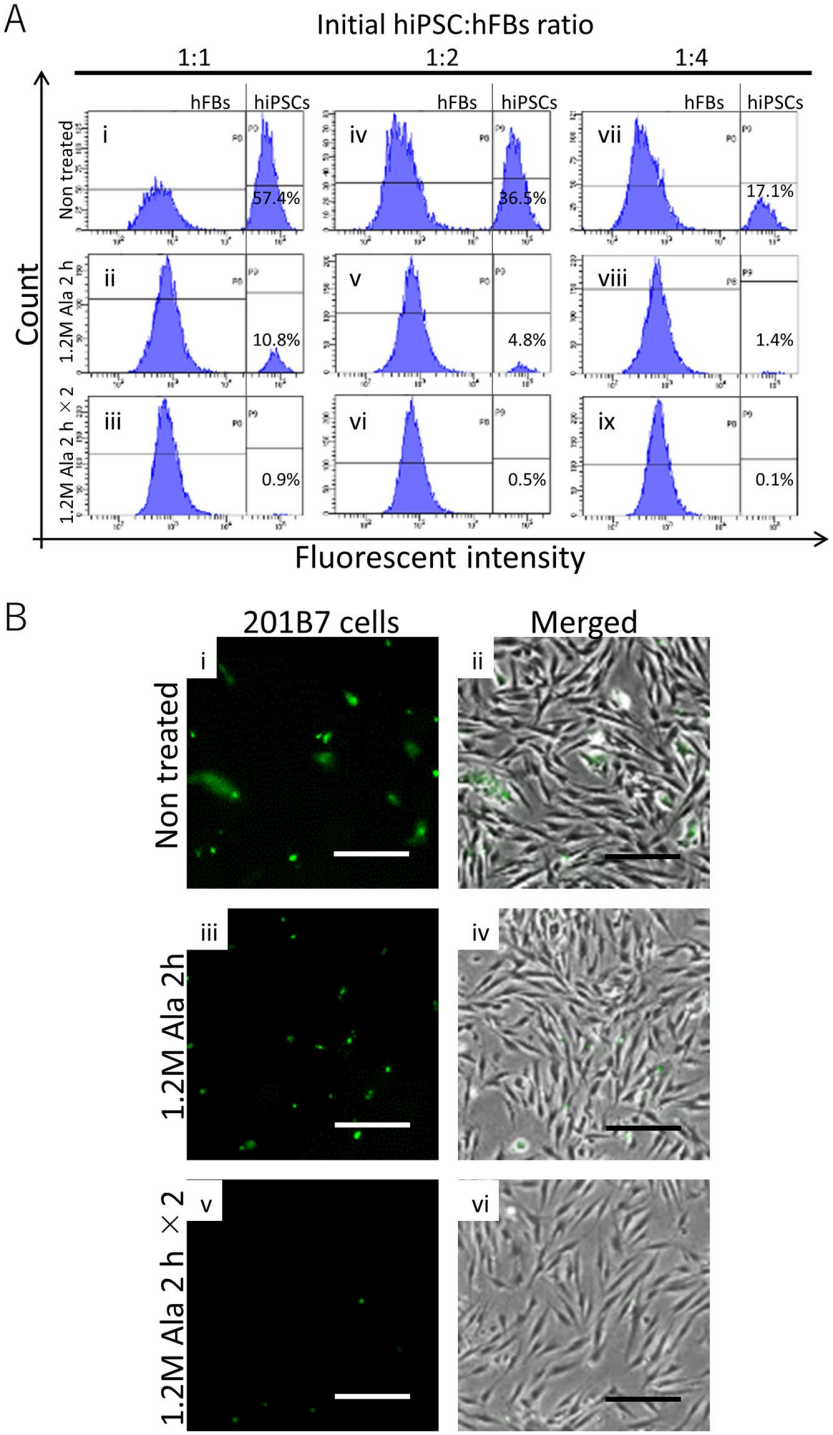
Selective elimination of hiPSCs co-cultured with hFBs in medium supplemented with L-alanine. 201B7 cells were fluorescently labeled with Cell Tracker Green. hFBs were seeded into a plate containing 201B7 cells and the medium was replaced with that containing 1.2 mol/L L-alanine. After 2 h, the cultivation medium was replaced with the normal medium. The cells were cultured for 12 h and flow cytometry data were acquired. (**A**) Flow cytometry analysis. Initial ratio of 201B7 cells to hFBs was 1:1 (i–iii), 1:2 (iv–vi), 1:4 (vii–ix). (i,iv and vii) Non-treated. (ii,v and viii) Single treatment. (iii,vi and ix) Repetitive treatment. (**B**) Images of cell before their collection for flow cytometry analysis. (i,iii and v) 201B7 cells stained with Cell Tracker Green. (ii,iv and vi) Merged images of phase-contrast images and the images in i, iii and v. (i and ii) Non-treated. (iii and iv) Single treatment. (v and vi) Repetitive treatment. Green: undifferentiated 201B7. Scale bar, 50 µm.

Without any treatment, 57.4%, 36.5%, and 17.1% of 201B7 cells were observed in 1:1, 1:2, and 1:4 condition, respectively (Fig. 3A-i,-iv and -vii). After treatment, the number of 201B7 cells decreased to 10.8%, 4.8%, and 1.4% in 1:1, 1:2, and 1:4 condition, respectively (Fig. 3A-ii,-v and -viii). By repetitive treatment, the percentage of 201B7 cells reduced to 0.9%, 0.5%, and 0.1% in 1:1, 1:2, and 1:4 condition, respectively (Fig. 3A-iii,-vi and -ix). As shown in Fig. 3B, the number of colonies of 201B7 cells decreased with an increase in the treatment time.

### Effects of substrates and temperature on the viability of hiPSCs

**Figure 4:**
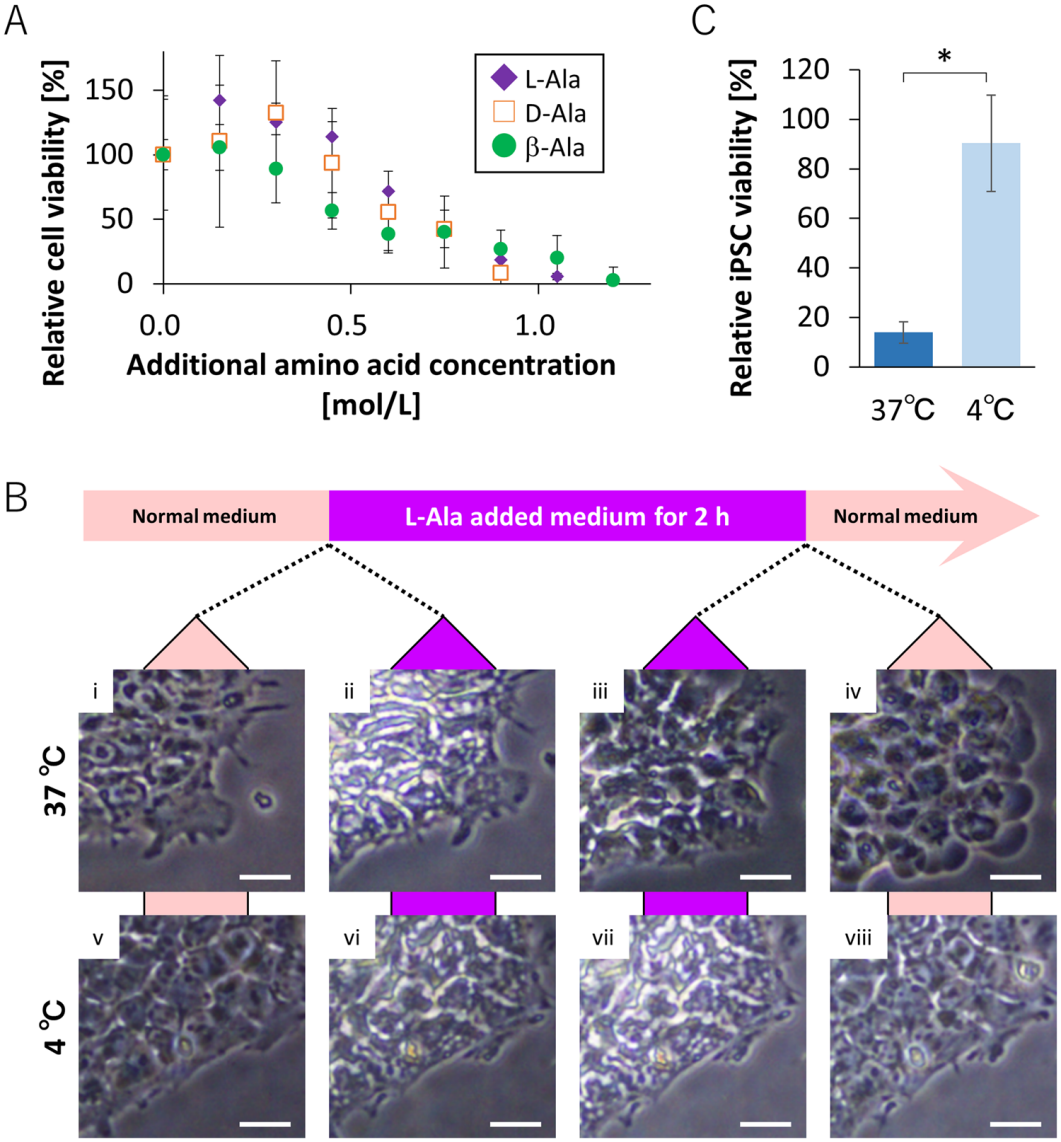
Effects of substrates and temperature on the death of iPSCs. (**A**) Viability of cells treated with L-alanine (filled purple diamond), D-alanine (empty orange square), and β-alanine (filled green circle) for 2 h. (**B**) Images of 201B7 cells before and after treatment with 1.2 mol/L L-alanine (i,ii,v and vi). Images before and after replacement of medium with the normal medium (iii, iv, vi and vii). Images of cells in the normal medium at 37 °C (i–iv) and 4 °C (v–viii). Scale bar, 50 µm. (**C**) Viability of 201B7 cells treated with medium supplemented with 1.2 mol/L of L-alanine for 2 h at 37 °C or 4 °C. The means ± SD of three experiments are shown. *p < 0.05.

To reveal the mechanism underlying the effect of L-alanine on the viability of hiPSCs, we treated cells with D-alanine and β-alanine. As shown in Fig. 4A, the decrease in the cell viability observed in the medium supplemented with D-alanine or β-alanine was similar to that observed with medium supplemented with L-alanine.

We investigated the viability of hiPSCs treated with medium supplemented with L-alanine at 37 °C and 4 °C (Movies S1–4). Figure 4B shows images of 201B7 cells at 37 °C (i-iv) and 4 °C (v-viii) treated under following conditions: before treatment with L-alanine (i and v), just after treatment (ii and vi), 2 h after treatment (iii and vii), and after the change of medium to normal medium (iv and viii). We observed that hiPSCs underwent dramatic shrinkage following treatment with medium supplemented with L-alanine at 37 °C (Fig. 4B-i,ii). After 2 h treatment, the volume of 201B7 cells was slightly recovered (Fig. 4B-ii,iii). When the medium was replaced with the normal medium, 201B7 cells drastically swelled (Fig. 4B-iii,iv). At 4 °C, on the other hand, cell shrinkage was observed following treatment with medium supplemented with L-alanine (Fig. 4B-v,vi), but 201B7 cells didn’t swelled drastically when the medium was replaced with the normal medium (Fig. 4B-vii–viii). Based on these results, we examined whether the treatment at 4 °C resulted in cell death and found no decrease in the viability of 201B7 cells following treatment with 1.2 mol/L L-alanine at 4 °C (Fig. 4C).

## Discussion

In this study, we found that the response to the treatment with high concentration of an amino acid was different between undifferentiated hiPSCs and differentiated cells, and these differences may be used for the selective elimination of undifferentiated hiPSCs. As L-alanine used in this study exists in human body, it may be non-toxic even when retained in differentiated cells after the treatment. Furthermore, L-alanine is mass-produced and generally used in cell culture medium. Taken together, the method suggested here may be safe, inexpensive, and efficient for the elimination of undifferentiated hiPSCs.

We used two types of undifferentiated iPSC cell lines, 201B7 cells and ehiPSCs. While 201B7 cells were established from cutaneous hFBs by lentivirus, ehiPSCs were derived from CD34-positive cord blood using episomal system. We investigated the responses of these two iPSC lines and hFBs to medium supplemented with L-alanine and found that the sensitivity of both iPSC lines to the medium with high concentration of L-alanine was higher than that of hFBs (Fig. 1B and C). These results suggest that our method is effective for several undifferentiated iPSC lines. Nonetheless, the sensitivities of two iPSC lines were slightly different (Fig. 1B and C). The viability of 201B7 cells was 38.3 ± 8.4% following treatment with 0.9 mol/L L-alanine for 2 h, while that of ehiPSCs was 6.5 ± 0.5%. These differences may be related to the original cell types or reprograming methods of iPSC lines. Thus, we believe that optimization of L-alanine concentration and treatment time may allow us to achieve higher efficiency for the selective elimination for each iPSC line.

Suspension cultures are promising sources for efficient mass production of hiPSCs^16–18^. Several methods using suspension culture have been reported for the differentiation of hiPSCs^19,20^. Thus, in the future, methods that allow elimination of residual undifferentiated hiPSCs in single-cell/cellular-aggregate suspension culture may be needed. In the present study, we treated 201B7 cells and hFBs in single-cell suspension culture with 1.2 mol/L L-alanine and confirmed that 201B7 cells were more sensitive than hFBs (Fig. 1D). These results suggest the application of this method not only for 2D adhesion culture but also for suspension culture and that it may play an important role for the mass production of hiPSCs.

Observation of iPSC-death would help elucidate the mechanism of different responses of hiPSCs and differentiated cells to the treatment with high concentration of L-alanine. As shown in Fig. 4B, hiPSCs underwent dramatic swelling following the change from medium with high level of L-alanine to the normal medium. After undergoing over swelling, hiPSCs were gradually stained with PI within a few hours. Thus, we found that the over swelling results in the rupture of plasma membrane functions of hiPSCs, leading to cell death. Since the dramatic swelling and death of hiPSCs were inhibited at 4 °C (Fig. 4B and C), the iPSC death observed in this study may be dependent on the mechanisms using intracellular energy, such as endocytosis^21^.

**Figure 5:**
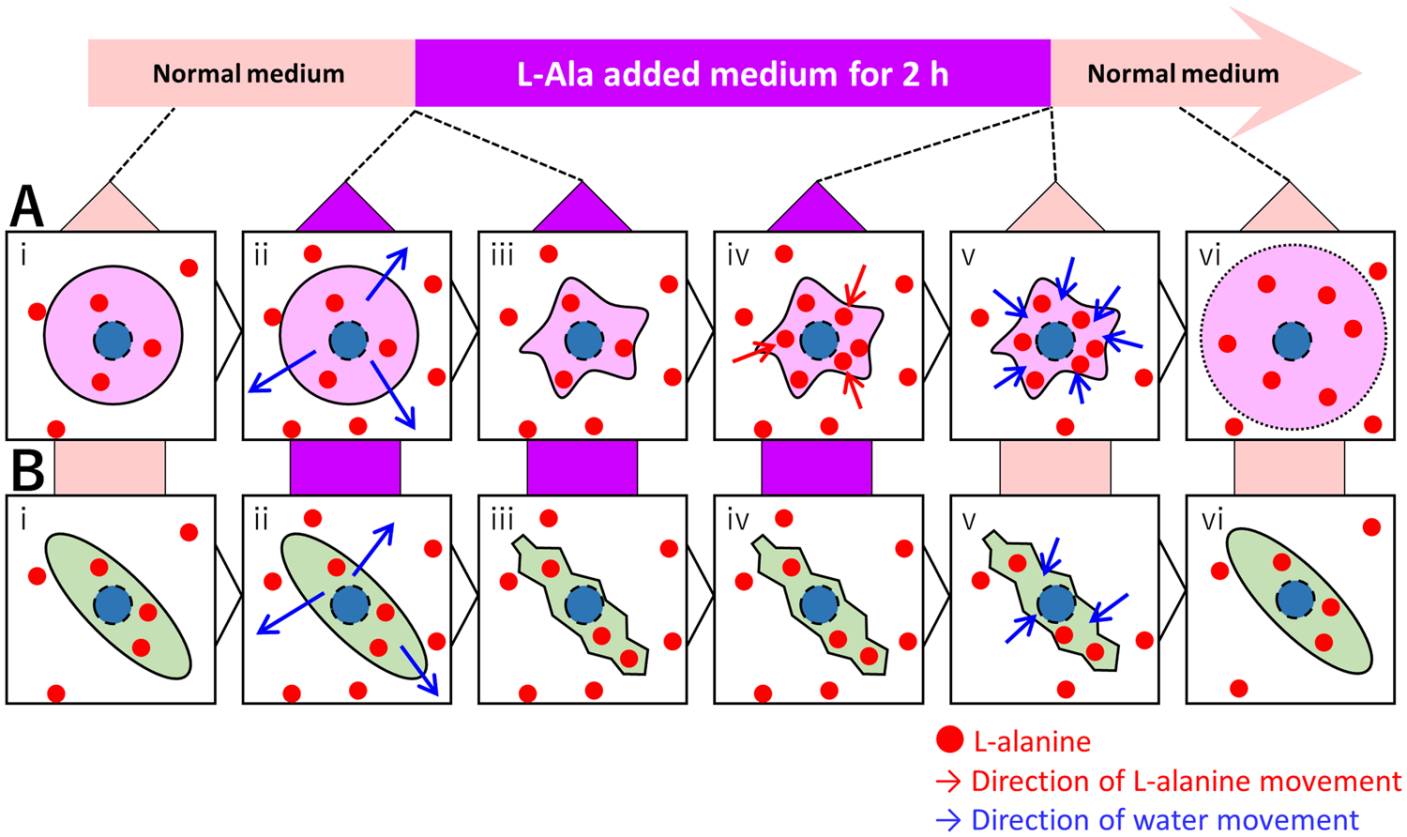
Schematic representation of the hypothesis of iPSC death and differentiated cell survival. (**A**) The hypothesis of iPSC death. (i) In normal medium, intracellular and extracellular solutes concentrations are equal. (ii) After changing the medium to that supplemented with high concentration of L-alanine, the hypertonic environment causes outward movement of water from the cell. (iii) Cells are shrunken. (iv) Some mechanism increases intracellular amino acid concentrations. (v) After changing the medium to normal medium, water diffuses into the cell because the normal media is relatively hypotonic. (vi) Cells are expanded and dead. (**B**) The hypothesis of differentiated cell survival. (i) In a normal medium, intracellular and extracellular solute concentrations are equal. (ii) After changing the medium to that supplemented with high concentration of L-alanine, the hypertonic environment causes outward movement of water from the cell. (iii) Cells are shrunken. (iv) Concentration of amino acids in the differentiated cell does not increase because of mechanisms preventing the excessive increase of intracellular L-alanine concentration. (v) After changing the medium to a normal medium, water diffuses into the cell because normal media are relatively hypotonic. (vi) Cell sizes are recovered.

From these observations, we hypothesized the iPSC death process to be as shown in Fig. 5A. The high concentration of L-alanine may result in the outward movement of water from the cells (Fig. 5A-i–iii). During the 2 h incubation in the high-L-alanine medium, iPSCs may have internalize L-alanine by mechanisms using intracellular energy, leading to hypertonic condition (Fig. 5A-iv). The replacement of medium containing L-alanine with normal medium may equilibrate the osmotic pressure (Fig. 5A-v). As a consequence, hiPSCs may uptake water, resulting in their swelling and consequent rupture of their plasma membranes (Fig. 5A-vi).

A drastic change in osmotic pressure was surmised to be one of the reasons for the selective elimination of undifferentiated cells. However, medium supplemented with NaCl, KCl, or D-glucose at high concentrations killed both hiPSCs and differentiated cells (Fig. 1E), whereas that supplemented with L-alanine killed hiPSCs but not differentiated cells. Although the mechanism behind this phenomenon remains to be elucidated, these results indicate that the selective elimination of hiPSCs is due not only to the drastic change in osmotic pressure, but also to the effects of biochemical or/and biological properties of L-alanine. They also strongly suggest that the differentiated cells have some mechanisms to endure the stress generated by the high–L-alanine medium.

Taken together, our results suggest that the differentiated cells have some mechanisms to prevent the excessive increase of intracellular L-alanine concentration during incubation in a high–L-alanine medium (Fig. 5B-iv); the differentiated cells might have mechanisms to avoid the excess uptake of L-alanine or to export intracellular excess L-alanine to the medium. This would circumvent the inward movement of the excess water (Fig. 5B-v), thereby preventing the swelling and consequent rupture of their plasma membranes (Fig. 5B-vi). Further analysis should be performed in the future to compare the mechanisms of L-alanine transport, such as endocytosis, exocytosis, and transporters, between hiPSCs and differentiated cells. In addition, experiments using other amino acids, such as glycine, serine, valine, cysteine, and phenylalanine would be valuable to clarify the mechanisms reported here.

For the practical application of this technology, it is necessary to clarify the suitability of this technology for other differentiated cells. So far, we have shown that this method is effective against FBs, SkMCs, and CMs (Figs 1 and 2). It should also be tested against other differentiated cells such as hepatocytes, blood cells, and mesenchymal stem cells. An interesting inquiry would be to clarify how and when hiPSCs gain tolerance during the differentiation process. As shown in Figs 1 and 2, human iPSCs-derived differentiated cells, iCMs and iFLCs had significantly higher tolerance against the high concentration L-alanine supplemented medium than undifferentiated iPSCs. Thus, future studies should investigate the relation between differentiation stages and sensitivity to high concentrations of amino acid supplemented medium.

In conclusion, we showed that high concentration of L-alanine added to the medium may selectively eliminate undifferentiated hiPSCs via a novel cell death process. This method may contribute to the development of a low-cost, safe, and practical method for regenerative medicine using hiPSCs to eliminate residual undifferentiated hiPSCs.

## Methods

### Cell Culture

201B7 cells^2^ were provided by RIKEN BRC through the Project for Realization of Regenerative Medicine and the National Bio-Resource Project of the MEXT, Japan. 201B7 cells and ehiPSC (A18945, Thermo Fisher scientific, USA) were maintained in Stemfit AK02N (Ajinomoto, Japan) in 25 cm^2^ cell culture flask (690–175; Greiner Bio-One, Austria) coated with 0.5 µg/cm^2^ laminin-511 E8 (iMatrix-511, 381–07363; Wako, Japan). The undifferentiated state of the hiPSCs was confirmed using rBC2LCN-FITC (Wako, Japan). hFBs (KF-4109; Kurabo, Japan) were cultivated in a 75 cm^2^ cell culture flask containing Dulbecco’s modified Eagle’s medium (DMEM; 08458–16, Nacalai Tesque, Japan) supplemented with 10% fetal bovine serum (FBS; Life Technologies, USA) and 1% penicillin-streptomycin (PS; Life Technologies). hSkMCs (CC-2561, Lonza, Switzerland) were cultivated in a 25 cm^2^ cell culture flask containing Skeletal Muscle Cell GM (C-39360, Promo Cell, Germany). iCMs (RCDC001N, ReproCell, Japan) were cultured according to the manufacturer’s instructions.

### Preparation of medium supplemented with L-alanine

A total of 1.11 g (0.012 mol) L-alanine (010–01042; Wako) was dissolved in 10 mL AK02N and filtered for sterilization. We defined this solution as the medium supplemented with 1.2 mol/L L-alanine, which was diluted to optimal concentrations. pH of the medium supplemented with 0 and 1.2 mol/L L-alanine were 7.40 ± 0.10 and 7.27 ± 0.06 respectively. Osmotic pressure of the medium supplemented with 0 and 0.6 mol/L L-alanine were 269.33 ± 1.53 and 958.33 ± 57.27 mOsm/L (OM-819, bio medical science, Japan).

### Cell viability assay

Both hiPSCs or hFBs were seeded (1 × 10^4^ cells/well) in 200 µL Stemfit AK02N medium containing 10 µM ROCK inhibitor (Y-27632, Wako) in 96-well plates (TR5003, True Line, USA) coated with 0.5 µg/cm^2^ laminin-511 E8. After 24 h, the cultivation medium was replaced with Stemfit AK02N (a normal medium) to remove ROCK inhibitor. On the next day, the culture medium in 96-well plates was replaced with 100 µL medium containing L-alanine. The plates were incubated for different time points at 37 °C or 4 °C. After incubation, the cultivation medium was replaced with the normal medium. On the next day, cell viability was determined using cell counting kit-8 (347–07621; Dojindo, Japan) following the manufacturer’s instructions.

To investigate the effects of L-alanine on cells in suspension culture, the cells were suspended in normal medium or medium supplemented with 1.2 mol/L L-alanine and incubated at 37 °C in 5% CO_2_ incubator. Both medium contained 10 µM ROCK inhibitor. After 2 h, the cells were collected by centrifugation at 160 × *g* for 5 min and resuspended in normal medium. The cells were seeded at 1 × 10^4^ cells/well in 200 µL AK02N medium containing ROCK inhibitor in laminin-511 E8-coated 96-well plate. On the following day, relative cell viability was determined using cell counting kit-8 and the equation below (1).

1 $${V}_{C}=\frac{{A}_{S}-{A}_{B}}{{A}_{C}-{A}_{B}}\times 100$$

where V_C_ is the relative cell viability, A_S_ is the absorbance of sample, A_B_ is the absorbance of blank sample, and A_C_ is the absorbance of positive control (positive control is untreated sample).

### Quantification of the selective elimination ability of medium supplemented with L-alanine

We fluorescently labeled iPSCs with 5 µM Cell Tracker Green CMFDA Dye (C2925, Thermo Fisher Scientific, USA) in AK02N for 30 min at 37 °C. Following incubation, varying number of stained iPSCs (5 × 10^5^, 2.5 × 10^5^, and 1.2 × 10^5^ cells) were seeded with 1 mL AK02N medium in 0.5 µg/cm^2^ laminin-511 E8-coated six-well plates. After 12 h, hFBs were seeded (5 × 10^5^ cells/well) and co-cultured with 201B7 hiPSCs for 12 h. The medium was replaced with 1 mL AK02N medium or medium supplemented with 1.2 mol/L L-alanine for 2 h. Following incubation, the cultivation medium was replaced with normal medium and the cells incubated at 37 °C for 12 h. The cells were dissociated into single cells by treatment with 0.5 × TrypLE Select for 4 min at 37 °C, followed by their collection using a cell scraper. The collected cells were centrifuged at 1,500 × *g* for 1 min and resuspended in PBS containing 1% bovine serum albumin BSA. These cells were analyzed using a flow cytometer (FACS Canto II; BD Biosciences, USA).

### Differentiation of hiPSCs

Differentiated hiPSC progeny (hFB-like cell; iFLC) were derived from hiPSC by spontaneous differentiation according to Alekseenko’s method^15^ via embryoid body (EB) formation. hiPSCs 201B7 cells were suspended in polystyrene dish (SH90-15, ASAHI GLASS, Japan) containing DMEM with 10% FBS, 1% PS, and 10 µM ROCK inhibitor. Within 14 days, cystic EBs were formed. EBs were transferred into tissue culture dishes containing same medium. Cells were subcultured once a week at a split ratio of 1:3. After 2–3 passages, the culture comprised morphologically homogenous iFLCs. Undifferentiated iPSCs and iFLCs were fluorescently labeled with 5 µM Cell Tracker Green CMFDA dye (C2925, Thermo Fisher Scientific, USA) in AK02N and 5 µM Cell Tracker Orange (C34551, Thermo Fisher Scientific) in DMEM for 30 min at 37 °C, 5% CO_2_ incubator. After labeling, the stained iPSCs (1 × 10^5^ cells) were seeded in 1 mL normal medium (Stemfit AK02N) in 0.5 µg/cm^2^ laminin-511 E8-coated six-well plates. After 12 h, labeled iFLCs were seeded (5 × 10^5^ cells/well) and co-cultured with 201B7 hiPSCs for 24 h. Following incubation, the medium was replaced with 1 mL normal medium or medium supplemented with 1.2 mol/L L-alanine. After 2 h, the cultivation medium was replaced with normal medium and cells were incubated at 37 °C, for 24 h, followed by their observation under a fluorescence microscope (BZ-X700, Keyence, Japan).

### Staining of dead cells

We seeded hiPSCs in 0.5 µg/cm^2^ laminin-511 E8-coated 35 mm dish (3000–035, ASAHI GLASS) with AK02N medium. After 5 days, iPSC colonies were formed. hiPSCs were exposed to normal medium or medium supplemented with 1.2 mol/L L-alanine for 2 h. Following incubation, the medium was replaced with normal medium containing 10 µg/mL PI (347–07881, Dojindo) and the cells were observed under a fluorescence microscope (LCV 110, Olympus, Tokyo).

### Statistical analysis

Date are presented as mean values and standard deviation (SD). Student’s *t*-test was used for evaluating statistical significance for comparison. A value of p < 0.05, p < 0.01, and p < 0.005 indicated statistical significance.

## Electronic supplementary material

Supplementary figure

Movie S1

Movie S2

Movie S3

Movie S4

## References

[1] Thomson JA (1998). Embryonic stem cell lines derived from human blastocysts. Science.

[2] Takahashi K (2007). Induction of Pluripotent Stem Cells from Adult Human Fibroblasts by Defined Factors. Cell.

[3] Medvedev SP, Shevchenko AI, Zakian SM (2010). Induced Pluripotent Stem Cells: Problems and Advantages when Applying them in Regenerative Medicine. Acta Naturae.

[4] Ben-David U, Benvenisty N (2011). The tumorigenicity of human embryonic and induced pluripotent stem cells. Nat. Rev. Cancer.

[5] Rodrigues GMC, Rodrigues CAV, Fernandes TG, Diogo MM, Cabral JMS (2015). Clinical-scale purification of pluripotent stem cell derivatives for cell-based therapies. Biotechnol. J..

[6] Yagyu S, Hoyos V, Del Bufalo F, Brenner MK (2015). An Inducible Caspase-9 Suicide Gene to Improve the Safety of Therapy Using Human Induced Pluripotent Stem Cells. Mol. Ther..

[7] Matsumoto R (2016). Plasma-activated medium selectively eliminates undifferentiated human induced pluripotent stem cells. Regen. Ther..

[8] Ben-David U, Nudel N, Benvenisty N (2013). Immunologic and chemical targeting of the tight-junction protein Claudin-6 eliminates tumorigenic human pluripotent stem cells. Nat. Commun..

[9] Tateno H (2015). Elimination of Tumorigenic Human Pluripotent Stem Cells by a Recombinant Lectin-Toxin Fusion Protein. Stem Cell Reports.

[10] Tohyama S (2013). Distinct Metabolic Flow Enables Large-Scale Purification of Mouse and Human Pluripotent Stem Cell-Derived Cardiomyocytes. Cell Stem Cell.

[11] Schriebl K (2012). Selective removal of undifferentiated human embryonic stem cells using magnetic activated cell sorting followed by a cytotoxic antibody. Tissue Eng. Part A.

[12] Ben-David U (2013). Selective elimination of human pluripotent stem cells by an oleate synthesis inhibitor discovered in a high-throughput screen. Cell Stem Cell.

[13] Lee MO (2013). Inhibition of pluripotent stem cell-derived teratoma formation by small molecules. Proc. Natl. Acad. Sci..

[14] Mao D (2017). A Synthetic Hybrid Molecule for the Selective Removal of Human Pluripotent Stem Cells from Cell Mixtures. Angew. Chem. Int. Ed. Engl..

[15] Alekseenko LL (2012). Heat shock induces apoptosis in human embryonic stem cells but a premature senescence phenotype in their differentiated progeny. Cell Cycle.

[16] Lei Y, Schaffer DV (2013). A fully defined and scalable 3D culture system for human pluripotent stem cell expansion and differentiation. Proc. Natl. Acad. Sci. USA.

[17] Lucendo-Villarin B, Rashidi H, Cameron K, Hay DC (2016). Pluripotent stem cell derived hepatocytes: using materials to define cellular differentiation and tissue engineering. J. Mater. Chem. B.

[18] Otsuji TG (2014). A 3D Sphere Culture System Containing Functional Polymers for Large-Scale Human Pluripotent Stem Cell Production. Stem Cell Reports.

[19] Pettinato G (2016). Scalable Differentiation of Human iPSCs in a Multicellular Spheroid-based 3D Culture into Hepatocyte-like Cells through Direct Wnt/β-catenin Pathway Inhibition. Sci. Rep..

[20] Shimojo D (2015). Rapid, efficient, and simple motor neuron differentiation from human pluripotent stem cells. Mol. Brain.

[21] De Figueiredo RC, Soares MJ (2000). Low temperature blocks fluid-phase pinocytosis and receptor-mediated endocytosis in Trypanosoma cruzi epimastigotes. Parasitol. Res..

