# Supplementary material for: Selective Elimination of Human Induced Pluripotent Stem Cells Using Medium with High Concentration of L-Alanine
Source: Sci Rep. 2018 Aug 20;8:12427. doi: 10.1038/s41598-018-30936-2 (PMC6102287; doi:10.1038/s41598-018-30936-2)
Supplement: Supplementary file 1 — Supplementary figure [file 41598_2018_30936_MOESM1_ESM.pdf]

Supplementary Information

Selective Elimination of Human Induced Pluripotent Stem Cells Using Medium with High Concentration of L-Alanine

Takunori Nagashima[1], Kazunori Shimizu[1,*], Ryo Matsumoto[1], Hiroyuki Honda[1,2]

[1]*Department of Biomolecular Engineering, Graduate School of Engineering, Nagoya University, Nagoya, Japan*
[2]*Innovative Research Center for Preventive Medical Engineering, Nagoya University, Nagoya, Japan*

*Correspondence: shimizu@chembio.nagoya-u.ac.jp

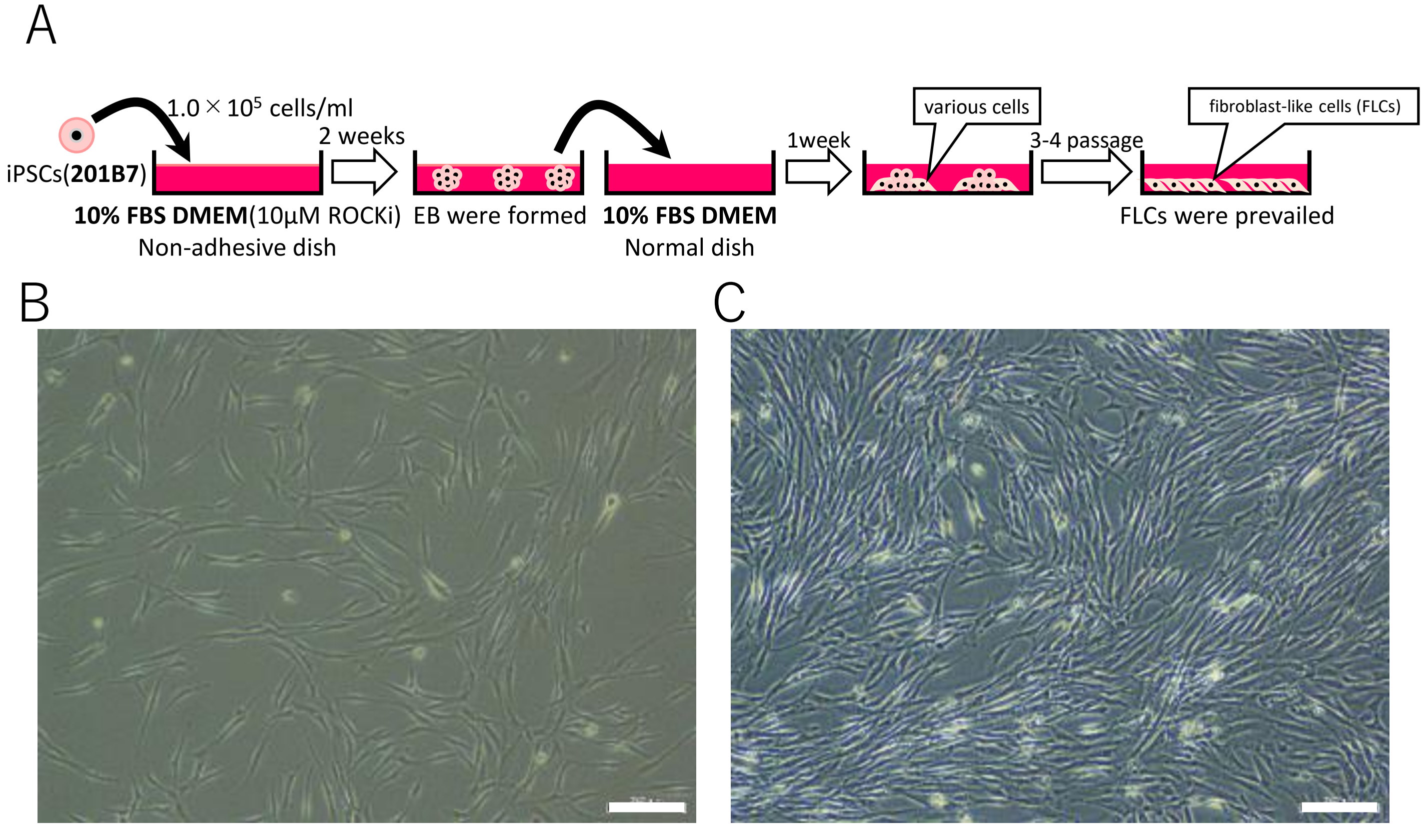

Figure S1. Preparation of hiPSC-derived fibroblast-like cells (iFLCs)
(A) Schematic representation of the differentiation procedure of 201B7 cells.
(B) Phase-contrast image of primary human fibroblasts.
(C) Phase-contrast image of iFLCs derived from 201B7 cells.
Scale bar, 200 µm.

Supplementary Movies

Videos were taken by optical microscope (CKX53, Olympus, Japan) and digital camera (DP21, Olympus). The timer in the video is written as mm:ss.

Movie S1. Shrinkage of hiPSCs following treatment with medium supplemented with L-alanine at 37 ° C
Related to Figure 4Bi-ii.

Movie S2. HiPSCs swelled drastically when the medium was replaced with the normal medium at 37 ° C
Related to Figure 4Biii-iv.

Movie S3. Shrinkage of hiPSCs following treatment with medium supplemented with L-alanine at 4 ° C
Related to Figure 4Bv-vi.

Movie S4. HiPSCs didn’t swelled drastically when the medium was replaced with the normal medium at 4 ° C
Related to Figure 4Bvii-viii.
